# Setting new research in the context of previous research: some options

**DOI:** 10.1136/bmjebm-2023-112300

**Published:** 2023-06-24

**Authors:** Paul Glasziou, Mark Jones, Mike Clarke

**Affiliations:** 1 Instiute for Evidence Based Heathcare, Bond University, Robina, Queensland, Australia; 2 Northern Ireland Methodology Hub, Queen's University Belfast, Belfast, UK

**Keywords:** Systematic Reviews as Topic

## Introduction

If a new clinical trial is to be justifiable both scientifically and ethically it should be designed in the light of an assessment of relevant previous research, ideally a systematic review. When its findings are reported, these should be set in the context of updated reviews of other, similar research.^1^

When reading the report of a new controlled trial, interpretation will be greatly aided if the Discussion sets the results in the context of the results from available similar research.^1^ ^2^ Indeed, the CONSORT statement^3^ (item 22) suggests including ‘a formal systematic review in the results or discussion section of the report’. However, CONSORT observes that ‘Such synthesis may be judged impractical for trial authors, but it is often possible to quote a systematic review of similar trials.’ For practical reasons, most authors are likely to choose the latter option, but this still leaves the reader to mentally weigh up the previous systematic review results and the new trial results. We have considered whether there might be better options and aimed to enumerate them here.

**Figure 1:**
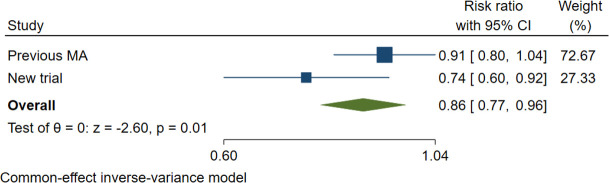
Example of how new trial findings can be visualised in the context of previous research.

As an example, consider a meta-analysis^4^ of lower vs versus usual targets for blood pressure lowering containing 6 trials and 41 491 patients which found an overall relative risk (RR) of 0.91 with 95% CI 0.80 to 1.04 and a new trial^5^ with 8511 patients with an RR 0.74; 95% CI 0.60 to 0.92. What do these mean together? The figure 1 shows these results as well as the combined estimate. The addition of the new trial has increased the effect size in the meta-analysis from a 9% relative reduction to a 14% relative reduction and the CI of the new estimate now excludes the null result (RR: 1.00), so is statistically significant.

Without this visual information in figure 1, most readers will be unable to mentally envisage what the implications of the new trial findings are.

## The methods

To address the issue of setting new trial findings in the context of previous research, we suggest including in the trial’s Discussion section a statement such as:

A recent systematic review found that [RESULTS]; adding the results of our new trial to this meta-analysis would change the effect to [NEW RESULTS].

This could place the new trial’s findings in the context of previous research without conducting a new systematic review ‘from scratch’. How might this be done? We present three alternative methods.

### Method 1: updated review

Conduct an updated systematic review. For readers, the ideal ‘Discussion’ would include an updated and up-to-date meta-analysis. Examples of this are the Discussion sections of the ISIS-1 trial of beta-blockers for myocardial infarction,^6^ and the TACT trial of taxanes for breast cancer,^7^ both of which include a ‘forest plot’ incorporating the result of the new trial with the results of the previous trials.^2^ However, no search strategies are provided, so it is unclear whether these updated meta-analyses include all the relevant trial evidence. Although an updated systematic review to gather all this evidence is possibly the best option methodologically, an important limitation is that it could take months of additional work, so would generally need to be conducted in parallel with the final stages of the new trial and such work might be outside the resources and expertise of many current clinical trial teams.

### Method 2: add to forest plot

Add the new trial result to the previous systematic review. Rather than do a complete systematic review, an easier option would be to add the results of the new trial—if eligible—to a previous meta-analysis. This only requires extracting the summary results for each trial from the previous meta-analysis, adding those for the new trial, and combining the results. This is particularly easy for Cochrane reviews because of the facility in the Cochrane Library to download the RevMan analysis dataset for every Cochrane review (from the ‘Download statistical data’ link under Contents). This allows the new trial data to be added to the meta-analysis in the Cochrane review to generate a new forest plot. Unfortunately, few people know or use this facility in the Library, and hence it should be better promoted. This updating facility was a feature of the Oxford Database of Perinatal Trials, the precursor of the Cochrane Library.

For non-Cochrane reviews, more effort would be required to extract the primary data from the previous meta-analysis, enter it into statistical software such as RevMan, and redo the analysis.

### Method 3: add to summary effect

Add the new trial result to the previous summary results (‘diamond’). Adding the current trial to the previous summary result is illustrated in Figure 1. This requires even less effort because all that is needed is that summary results and not the results of each trial in the previous meta-analysis. However, with this option, it is not possible to redo a random effects meta-analysis; unlike fixed effects models, random effects models also require estimation of the statistical heterogeneity for the study weightings which in turn requires the results of all individual studies to enable re-estimation of the statistical heterogeneity. Given the large number of systematic reviews and meta-analysis that now exist^8^ for some topics and the possibility that the new trial would be eligible for several of these, this option may require decisions around which previous systematic review or meta-analysis to use, how out-of-date it might be, and how many other relevant trial results may have been published since the systematic review was last updated.

For both methods 2 and 3, the trial authors should assess and report on the quality and recency of the referenced systematic review, and should consider how well their trial question (‘PICO’) fits with those in the systematic review. Ideally they should spell out any implications for the quality of evidence. These assessments may require assistance from an experience systematic reviewer.

## Discussion

There have been repeated calls for new trials to be set in the context of previous similar studies or a previous systematic review. We have provided three possible options, all of which have limitations, but all are preferred to not providing such context to trial readers or of using only a selection of the previous research in the Discussion section. Method 3 is the simplest option but is less appropriate in the presence of substantial statistical heterogeneity and, given that we are not aware of a way to combine the new trial result and the previous meta-analysis diamond that would take account of heterogeneity, we recommend method 2 if a new meta-analysis is judged to be likely to be meaningful. Method 1 is perhaps the most methodologically sound option, but it may require substantial work, expertise and time, unless it has been planned in advance.

However, if, as should have been the case, one or more systematic reviews had provided the justification for embarking on the new trial, this need not be a massive challenge. An impressive early application of this principle is illustrated by the report of the (first) ISIS trial.^6^

**Excerpt from abstract**

Systematic review of fatal and of non-fatal events in ISIS-1 and in all other randomised trials of iv beta-blockade reinforces the suggestion that treatment reduces mortality in the first week by about 15%, but with a rather less extreme effect in days 0–1 than was observed in ISIS-1 alone. It also provides highly significant (2p<0.0002) evidence of an effect on the combined end-point of death, arrest, or reinfarction, suggesting that treatment of about 200 patients would lead to the avoidance of 1 reinfarction, 1 arrest, and 1 death during days 0–7. ISIS-1 suggests these early gains will persist.

At the time, this was described as a ‘lengthy tail piece’ by the editorial that accompanied the ISIS-1 report, when the author made it clear that he would not allow such ‘lengthy tail pieces’ to become routine in the Lancet.^9^ We would argue that such tail pieces are vital to the reader’s interpretation of any new trial.

An early intention of the Cochrane Collaboration was that all Cochrane reviews would be updated continually with the addition of new evidence^10^ but the work entailed has limited this to every few years. However, there is currently a move towards living systematic reviews which are updated as new evidence emerges.^10 11^ These could provide a further potential option to setting new trial findings in the context of previous research, perhaps by publishing a new version of the living systematic review at the same time as the new trial but it may take some time before living systematic reviews are in wide use and this would require coordination between the researchers responsible for the new trial and those responsible for the living systematic review.^12^ Though we have focused on systematic review of clinical trials, the three methods described here are relevant for systematic reviews of all types of studies, though would need some minor adaptations. Finally, we note that with living systematic reviews and our three methods, caution is needed because of the risk of ‘false positives’ from multiple sequential testing.^13^
